# A Comprehensive Assessment of the Safety of *Blautia producta* DSM 2950

**DOI:** 10.3390/microorganisms9050908

**Published:** 2021-04-23

**Authors:** Xuemei Liu, Weiling Guo, Shumao Cui, Xin Tang, Jianxin Zhao, Hao Zhang, Bingyong Mao, Wei Chen

**Affiliations:** 1 State Key Laboratory of Food Science and Technology, Jiangnan University, Wuxi 214122, China; 6180112042@stu.jiangnan.edu.cn (X.L.); weilingguo2021@163.com (W.G.); xintang@jiangnan.edu.cn (X.T.); zhaojianxin@jiangnan.edu.cn (J.Z.); zhanghao61@jiangnan.edu.cn (H.Z.); chenwei66@jiangnan.edu.cn (W.C.); 2 School of Food Science and Technology, Jiangnan University, Wuxi 214122, China; 3 National Engineering Research Center for Functional Food, Jiangnan University, Wuxi 214122, China

**Keywords:** *Blautia producta*, safety assessment, probiotics, virulence genes, antibiotic resistance, acute toxicity, mice

## Abstract

In recent years, *Blautia* has attracted attention for its role in ameliorating host diseases. In particular, *Blautia producta* DSM 2950 has been considered a potential probiotic due to its ability to mitigate inflammation in poly(I:C) induced HT-29 cells. Thus, to promote the development of indigenous intestinal microorganisms with potential probiotic function, we conducted a comprehensive experimental analysis of DSM 2950 to determine its safety. This comprised a study of its potential virulence genes, antibiotic resistance genes, genomic islands, antibiotic resistance, and hemolytic activity and a 14-day test of its acute oral toxicity in mice. The results indicated no toxin-related virulence genes in the DSM 2950 genome. Most of the genomic islands in DSM 2950 were related to metabolism, rather than virulence expression. DSM 2950 was sensitive to most of the tested antibiotics but was tolerant of treatment with kanamycin, neomycin, clindamycin, or ciprofloxacin, probably because it possessed the corresponding antibiotic resistance genes. Oral acute toxicity tests indicated that the consumption of DSM 2950 does not cause toxic side effects in mice. Overall, the safety profile of DSM 2950 confirmed that it could be a candidate probiotic for use in food and pharmaceutical preparations.

## 1. Introduction

It is estimated that there are 10–100 trillion microorganisms in the human gastrointestinal tract, including a variety of bacteria, viruses, archaea, and eukaryotes, with more than 90% being members of the phyla Firmicutes or Bacteroidetes [1]. These microorganisms establish a symbiotic relationship with their host, and those that perform specific health-enhancing biological functions when ingested in adequate concentrations are known as probiotics [2]. Traditional probiotics, such as *Lactobacillus* spp. and *Bifidobacterium* spp., produce metabolites such as short-chain fatty acids, bacteriocins, and antimicrobial peptides, which can reduce inflammation, protect against pathogenic microbial infections, increase the integrity of the intestinal epithelium, and modulate immunity to promote host health [3,4,5,6]. The development of next-generation sequencing technology has led to a more comprehensive and in-depth understanding of the role of gut bacteria in human health and disease [7]. Compared with traditional probiotics, newly identified native intestinal bacteria such as *Akkermansia*, *Bacteroides*, *Clostridium*, and *Blautia* have rapidly attracted more attention for their health-promoting and therapeutic utility [8,9].

As a genus of the *Lachnospiraceae* family, members of which are highly abundant in the human gut, *Blautia* has been of particular interest due to its ability to alleviate inflammatory and metabolic diseases and for its antibacterial activity against specific microorganisms [10,11]. Increased *Blautia* abundance is associated with a reduction in the lethality of graft-versus-host diseases and improved overall survival [12], whereas decreased *Blautia* abundance is associated with liver cirrhosis [13], inflammatory bowel disease, and colorectal cancer [14,15]. *Blautia* abundance is also significantly negatively correlated with visceral fat accumulation [16], and in recent years, it was demonstrated to play a direct role in the alleviation of host diseases. In particular, *B. producta* DSM 2950 reduces inflammation in HT-29 cells [17]. Similarly, *B. producta* SCSK, isolated from feces, secretes antibiotics that inhibit the growth of vancomycin-resistant *Enterococcus faecium* and may act as a probiotic. This could provide new insights into the prevention of infection and the spread of antibiotic-resistant conditionally pathogenic bacteria [18].

Lists are compiled of conventional probiotics (e.g., *Lactobacillus* spp., *Bifidobacterium* spp.) that are considered safe due to their long history of consumption, and these probiotics are widely used as ingredients or additives in foods [19]. However, *Blautia* lacks a history of consumption in the human diet, and thus, it and other microorganisms that may act as potential probiotics must be subjected to a preliminary safety assessment based on their intended use in food or medicine. Therefore, in this study, we evaluated the virulence and antibiotic resistance genes, the genomic islands, the antibiotic resistance, and the hemolytic ability of the *B. producta* DSM 2950 strain and assessed its 14-day acute oral toxicity in mice. This established a theoretical basis for the development and use of functional microorganisms with probiotic properties.

## 2. Materials and Methods

### 2.1. Bacterial Strains and Culture Conditions

*B. producta* DSM 2950 was purchased from the BeNa Culture Collection (Shanghai, China) and was anaerobically cultured at 37 °C in modified Gifu Anaerobic Medium (GAM) medium [20], which consisted of 1.0% (*m*/*v*) protease peptone, 0.3% soy peptone, 0.5% yeast extract, 0.22% beef extract, 1.35% digested serum, 0.12% liver extract, 0.8% glucose, 0.25% potassium dihydrogen phosphate, 0.3% sodium chloride, and 0.06% l-cysteine hydrochloride, at a final pH of 7.3 ± 0.1. Cultures were obtained for in vitro experiments during the early stationary phase. Cells were harvested for in vivo experiments by centrifugation (8000× *g*, 20 min) and washed twice in phosphate-buffered saline (PBS; pH 7.4, 10×, purchased from Sinopharm Chemical Reagent Co., Ltd. Shanghai, China). The cells were then resuspended in 10% skimmed milk and preserved at −80 °C.

### 2.2. Draft Genome Sequencing

After three generations of activation, 4 mL of DSM 2950 was inoculated into 100 mL of GAM medium for expansion. The resulting inoculated medium was subjected to anaerobic incubation for 10 h and then centrifuged at 8000× *g* for 10 min to obtained cell pellets. The draft genomes of the pellets were sequenced on an Illumina HiSeq X Ten platform (Majorbio BioTech Co., Ltd., Shanghai, China). The quality of sequencing fragments was controlled using FastQC software [21]. SOAPdenovo2 (Version 2.0) was used to assemble high-quality sequencing data and partial gaps were filled by GapCloser [22,23]. Gene annotation was performed using BLASTP (BLAST, Version 2.3.0) with reference to the Kyoto Encyclopedia of Genes and Genomes, nr, SWISS-PROT, Clusters of Orthologous Genes, Pfam, and Gene Ontology databases. The draft genome sequence has been submitted to the GenBank of the NCBI database (accession number SRR14193994).

### 2.3. Profile of Safety-Related Genes

Antibiotic resistance genes and virulence genes were identified by local BLASTP with reference to the Comprehensive Antibiotic Resistance Database (CARD, http://arpcard.Mcmaster.ca, Version 1.1.3, accessed on 19 April 2021) and the virulence factor database (VFDB, http://www.mgc.ac.cn/VFs/, Version 1.0, accessed on 19 April 2021), respectively. We only considered BLAST results exhibiting more than 30% identity and 70% coverage and used a cut-off e-value of less than 0.01 [24,25]. IslandViewer (http://www.pathogenomics.sfu.ca/islandviewer/resources/, Version 4.0, accessed on 19 April 2021) was used for the identification and visualization of possible genomic islands in the DSM 2950 genome to assess its potential pathogenicity [26].

### 2.4. Hemolytic Activity

The hemolytic activity of DSM 2950 was tested on GAM agar supplemented with 0.01% hemin, vitamin K1, and 5% ovine blood. The hemin and vitamin K1 were purchased from Sangon Biotech (Shanghai, China) Co., Ltd., and sterile defibrated sheep blood was purchased from Bkman Biotechnology (Changde, China) Co., Ltd. First, 100 μL of culture at an appropriate dilution was spread on the agar, which was then anaerobically incubated at 37 °C for 48 h. After incubation, the hemolytic activity was categorized as β-hemolysis (clear halos around colonies), α-hemolysis (greenish halos around the colonies), or γ-hemolysis (the absence of hemolysis). Staphylococcus aureus ATCC29213, which exhibits β hemolysis, was used as a positive control [27].

### 2.5. Antibiotic Susceptibility Test

According to the ISO 10,932 standard [28], the broth microdilution method was used to determine the resistance of DSM 2950 to 12 antibiotics [29] (streptomycin, neomycin, tetracycline, erythromycin, clindamycin, ampicillin, amoxicillin, trimethoprim, ciprofloxacin, chloramphenicol, rifampin, and vancomycin) with different antibacterial mechanisms. Briefly, a twofold series of antibiotic dilutions were prepared in deionized water to give final concentrations ranging from 0.032 to 2.048 μg/mL, and 100 μL of each dilution was added to a sterile 96-well plate. The strain cultures (100 μL) were also diluted in GAM medium and added to the antibiotic dilutions to give a final inoculum density of 3 × 10^5^ CFU/mL, and *Lactobacillus paracasei* ATCC 334 was used as a positive control. The plate was then subject to anaerobic incubation at 37 °C for 48 h. After incubation, the optical density was measured at 600 nm on a microplate reader, and the concentration of an antibiotic that inhibited 90% of DSM 2950 growth was denoted the minimum inhibitory concentration (MIC) [30]. With reference to the standard European Food Safety Authority (EFSA) guidelines and the recommendations of the European Commission’s Scientific Committee on Animal Nutrition, the cut-off values were used to determine the tolerance of microorganisms to the antibiotic: if an MIC value is less than or equal to the breakpoint value, a microorganism is considered to be sensitive to the antibiotic [31,32]. Three biological replicates of all experiments were performed.

### 2.6. Animals

Thirty male and female C57BL6/J mice (7 weeks old, 20 ± 2 g) were purchased from Charles River (Beijing, China) and were raised in specific pathogen-free conditions at room temperature (25 ± 2 °C) and under a 12-h light/dark cycle in the barrier facility of the Animal Center of Jiangnan University (Wuxi, China) [33]. All of the animals were fed standard chow and sterile water and were acclimatized for 1 week before the experiment began.

### 2.7. Acute Toxicity Experimental Design

The mice were divided into six groups: a control male group, a 10^9^ male group, a 10^10^ male group, a control female group, a 10^9^ female group, and a 10^10^ female group (Table S1). Each control group was treated via oral gavage with 200 μL of skimmed milk (10% *w*/*v*) per day, each 10^9^ group was treated via oral gavage with 200 μL of 5 × 10^9^ CFU/mL DSM 2950 per day, and each 10^10^ group was treated via oral gavage with 200 μL of 5 × 10^10^ CFU/mL DSM 2950 per day [34]. The experiment lasted for 14 days with a consistent daily gavage time. The protocol for this study was approved by the Ethics Committee of Jiangnan University, Wuxi, China (JN.No20200630c0300815[125]) and complied with the Directive of 2010/63/European Community. Throughout the experiment, mice behavior and body weights were observed and recorded, in addition to their food and water intake. At the end of the experiment, mice were anesthetized with pentobarbital sodium (30 mg/kg). The mice were sacrificed after collecting blood samples by cardiac puncture. The heart, liver, lung, kidney, spleen, and colon were collected for subsequent analyses.

### 2.8. Hematological Measurements and Biochemical Parameter Analysis

After the experiment, 50-μL blood samples were collected in anticoagulation tubes, and hematological parameters were measured using an automatic hematology analyzer (BC-5000, Mindray, Shenzhen, China) within 0.5 h of collection. Serum specimens were obtained by blood centrifugation (3000 rpm for 15 min at 25 °C). Biochemical parameters were quantified using a fully automated biochemical analyzer (BS-480) and the corresponding reagent kit (Marex, Shenzhen, China).

### 2.9. Organ Index

After the mice were sacrificed, the heart, liver, lung, kidney, spleen, and colon were weighed and the relative weights of these organs were determined.

An organ index (%) (heart, liver, spleen, kidney, and colon) was calculated as [organ weight (g)/body weight (g)] × 100.

### 2.10. Histology Study

Liver, kidney, spleen, and colon tissues were taken and immediately fixed for 24 h in 4% paraformaldehyde dissolved in 0.01 M PBS at a pH of 7.4. Then, after dehydration by treatment with serial dilutions of ethanol and xylene, the specimens were embedded in paraffin. A microtome was used to cut 5-μm-thick sections of the resulting embedded tissues, and these sections were stained with hematoxylin and eosin (H&E). Images of sections were captured by scanning with a Pannoramic MIDI Digital Slide Scanner (3D-Histech Co., Ltd., Budapest, Hungary).

### 2.11. Statistical Analysis

All the experiments were conducted in triplicate, and data were expressed as mean ± standard deviation. SPSS 22.0 statistical software (IBM Corporation, Chicago, IL, USA) was used for data analysis using single-factor analysis of variance between the groups with Tukey’s multiple comparisons test. *p* < 0.05 was considered statistically significant, while *p* < 0.01 was considered extremely statistically significant.

## 3. Results

### 3.1. Virulence Genes and Genomic Islands of DSM 2950

Based on the VFDB database, a total of 96 virulence genes were identified in DSM 2950. In general, the regulation of the virulence-associated genes and the nonspecificity of the virulence factors showed that DSM 2950 exhibited no specific pathogenicity. The predicted offensive virulence genes were related to the adhesion and secretion system. Bacterial adherence to and invasion of host tissue and cells may increase the pathogenicity of bacterial infections, such as urinary tract infections and infective endocarditis [35]. The predicted defensive virulence genes were mainly involved in the production of stress proteins and in anti-phagocytosis processes, and in capsule biosynthesis to evade the host immune system (Table 1) [36].

### 3.2. Genomic Islands of DSM 2950

Horizontal gene transfer (HGT) is a key way by which bacteria adapt to their environment. For example, genomic islands (GIs) are large chromosomal regions containing mobile elements (10–200 Kb) that can be relayed to other bacteria via HGT [37,38]. Ten GIs were identified in the genome of DSM 2950 (Table S2), with most being responsible only for metabolism, and these are there to maintain the genomic island, and are not acting as external toxin. Only two GI groups (GI 03 and GI 08) carried the gene for the toxin (toxin RelE and antitoxin). RelE belongs to the bacterial toxin–antitoxin (TA) system, which can stall ribosomes by cleaving mRNA in a translation-dependent manner to facilitate the adaption of cells to environmental stresses [39].

### 3.3. Antibiotic Resistance of DSM 2950

Table 2 shows the antibiotic susceptibility of DSM 2950, which was determined by the broth microdilution method. Based on experiments performed as per the ISO 10,932 standard and EFSA guidelines, DSM 2950 was sensitive to streptomycin, tetracycline, erythromycin, ampicillin, amoxicillin, trimethoprim, chloramphenicol, rifampin, and vancomycin. The resistance genes were identified by CARD annotation (Table 3), showing that DSM 2950 was highly resistant of a macrolide antibiotic, a fluoroquinolone antibiotic (such as ciprofloxacin), a clindamycin antibiotic (such as clindamycin), a glycopeptide antibiotic, and an aminoglycoside antibiotic (such as neomycin), which is consistent with our experimental results.

### 3.4. Hemolytic Activity

**Figure 1:**
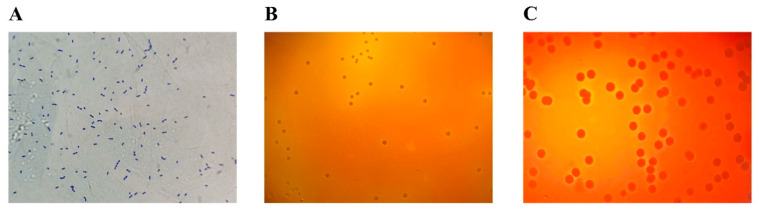
Morphology of *Blautia producta* DSM 2950. (**A**) Gram stain morphology of DSM 2950. (**B**) Hemolytic properties of DSM 2950. (**C**) Hemolytic properties of *Staphylococcus aureus*.

DSM 2950 was strictly anaerobic, gram-positive, and coccobacillus-shaped (Figure 1A). The colonies that grew on a plate containing 5% (*v*/*v*) ovine blood after anaerobic incubation at 37 °C for 48 h were circular, gray in color with a white-colored center, and convex with a smooth surface. *S. aureus* formed a clear β-hemolytic ring, but DSM 2950 had no hemolytic activity (γ-haemolysis) (Figure 1B,C).

### 3.5. Effects of DSM 2950 on Body Weight and Food and Water Intake

**Figure 2:**
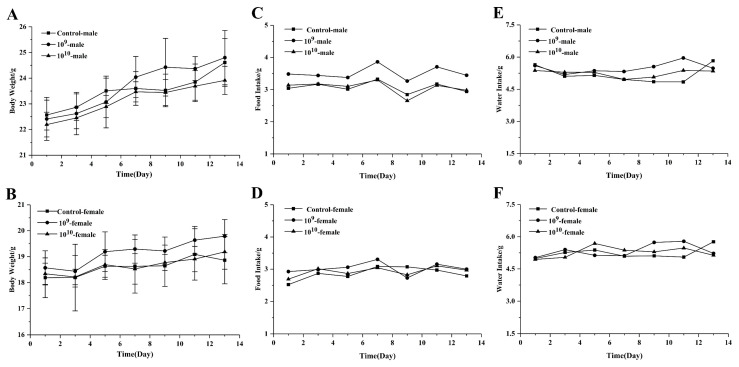
Effects of *Blautia producta* DSM 2950 on body weight and food and water intake. (**A**) Body weight of male mice. (**B**) Body weight of female mice. (**C**) Food intake of male mice. (**D**) Food intake of female mice. (**E**) Water intake of male mice. (**F**) Water intake of female mice.

During the 14-day acute toxicity test, the mice grew well, behaved normally, lost no hair, and all remained alive. In terms of the body weight of both male and female mice, no significant differences were observed between the 10^9^ and 10^10^ groups and the control group (Figure 2A,B). The daily food intake of mice was approximately 3 g and the water intake was approximately 5 g. No difference was detected in the food and water intake between the different groups (Figure 2C–F).

### 3.6. Effects of DSM 2950 on the Organ Index

**Figure 3:**
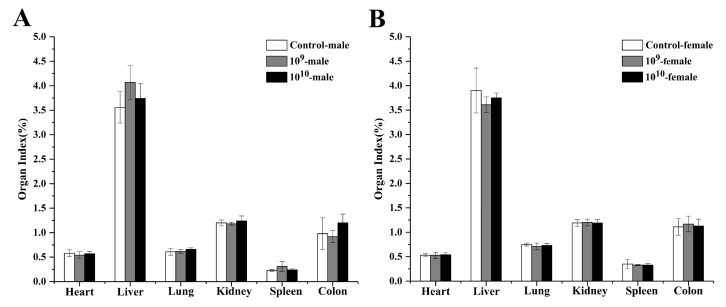
Effect of *Blautia producta* DSM 2950 on the organ index. (**A**) Organ index of male mice. (**B**) Organ index of female mice. *p* < 0.05 was considered statistically significant (*), while *p* < 0.01 was considered extremely statistically significant (**). The results indicated no significant differences among groups.

The results of organ index analyses are presented in Figure 3. No lesions were visible in the heart, liver, spleen, lungs, kidneys, or intestines of the mice. Compared with the control groups, the groups of mice treated with different doses of DSM 2950 showed no significant differences in the weights of various organs.

### 3.7. Effects of DSM 2950 on Biochemical Parameters

The effects of different doses of DSM 2950 strains on hematological parameters are shown in Table 4. In general, there were no significant differences in the number and proportion of blood cells between the control groups and the treatment groups (Tukey’s multiple comparisons test). In addition, there were no abnormalities in the blood biochemical parameters related to liver function and no significant differences in the values of these parameters between the control and treatment groups (Table 5).

### 3.8. Effects of DSM 2950 on Tissue Histology

**Figure 4:**
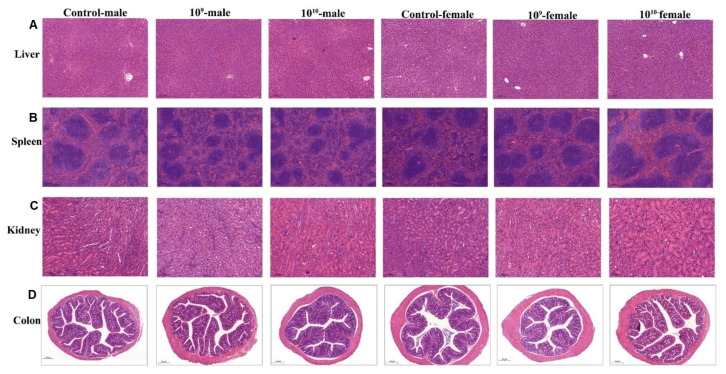
Representative photomicrographs of hematoxylin-and-eosin-stained sections of organ tissues of mice. (**A**) Liver tissue. (**B**) Spleen tissue. (**C**) Kidney tissue. (**D**) Colon tissue.

The H&E-stained liver, kidney, and spleen tissues of mice in the control groups and those in the different treatment groups are shown in Figure 4. It can be seen that DSM 2950 did not cause significant histopathological damage to the organs of mice. In particular, the structure of hepatic tissues in each group is distinguishable, and the hepatic sinusoids and hepatic cords are regularly arranged, with no significant hepatocyte degeneration, apoptosis or necrosis observable (Figure 4A); the kidney cells are well arranged and structurally intact, with clear nuclei and uniform cytoplasmic staining (Figure 4B); the splenic tissue is clear, with the red marrow and white marrow structurally intact and well defined, with the splenic cords and sinuses neatly aligned (Figure 4C); and the colon tissue has good morphology with a complete crypt structure enriched in goblet cells (Figure 4D).

## 4. Discussion

Commercial probiotics such as *Bifidobacterium* and *Lactobacillus* are usually isolated from traditional fermented foods and are regarded as safe due to their long history of consumption. However, some microbial species or strains that are dominant inhabitants of the human intestine and have specific efficacy, and are thus potential probiotics, must be subjected to a thorough safety assessment [40]. In recent years, it has been discovered that *Blautia* positively contributes to biotransformation, relieves inflammatory and metabolic diseases, and has antimicrobial activity against certain microorganisms. For example, *B. glucerasei* sp. nov. HFTH-1T produces extracellular enzymes that can rapidly hydrolyze plant glucose ceramides into functional substances with specific biological activities, such as ceramide, which may protect against colon cancer [41], and *B. obeum* A2-162 isolated from the human intestine can produce the novel lantibiotic nisin O, which has antibacterial activity against *Clostridium perfringens* [42]. In addition, in a cross-sectional study conducted in an obese population, we found that the abundance of *Blautia*, especially *B. luti* and *B. wexlerae*, was significantly lower in the intestinal flora of obese children than in that of non-obese children [43]. In addition, the abundance of *Blautia* in individuals with inflammatory pouch complications, compared to that in healthy individuals, decreased after ileal pouch anal anastomosis [44], and the abundance of *Faecalibacterium prausnitzii* and *Blautia* in the intestinal flora of patients with colitis-associated cancer [45]. DSM 2950 exerts its anti-inflammatory effects in HT-29 cells by decreasing poly(I:C) induced inflammatory transcription, which reduces the concentration of proinflammatory signaling molecules (IL-8, TNF, CXCL-10 and CXCL-11) [17]. Based on the above evidence and given the potential utility of DSM 2950 to relieve inflammation, we performed a comprehensive safety assessment to determine its suitability for human consumption. This comprised a systematic safety evaluation of DSM 2950 in genotypic and phenotypic assays and acute toxicity experiments to determine whether it is a novel probiotic that is beneficial to human health.

Substances that are generated by microorganisms and that contribute to their infectivity and cause specific host diseases are called virulence factors. Determining the pathogenicity of bacteria, which depends on their ability to use virulence factors, has recently become important for evaluating the safety of microorganisms [46]. For example, PhoP-PhoQ is an important two-component regulatory system in bacteria, as *PhoP* is a virulence factor that acts as a response regulator to help bacteria improve their adaptation to the external environment [47,48], whereas *FbpABC* and *HitABC* are virulence factors that are involved in iron-uptake systems and are ubiquitous in soil microorganisms [49]. β-hemolysins possess phospholipase C activity, which is conducive to diverse immune escape strategies of bacteria and provide the bacteria with access to nutrients to permit survival of pathogens [50]. Previous studies have shown that the presence of α-hemolysin (Hla) in *Staphylococcus aureus* could have harmful effects on host cells through accelerating cytotoxic lysis and the secretion of pro-inflammatory mediators and cytokines [51,52]. However, Paul et al. [53] found that Hla could promote the resolution of infectious inflammation by stimulating the production of specialized pro-resolving mediators to exert beneficial functions. Many pathogenic strains could produce cytolysin, which causes septicemia and serious wound infection in disease [54,55]. In fact, the production of cytolysin is a complex process and is influenced by many factors, including internal factors (genes) and external factors (temperature, humidity, and pH) [56]. For example, specific environmental conditions or sensitive manners could regulate the expression of the cytolysin [57], so the hemolysin/cytolysin genes identified in strains does not fully represent if it is pathogenic.

In another example, the Dot/Icm secretion system affects cell functions such as cytoskeletal reorganization and transcriptional regulation by secreting different types of virulence effectors [58]. Similarly, *Listeria monocytogenes* generates *FbpA*, which is a virulence factor that functions as an adhesion protein and is responsible for its infectivity and pathogenicity [59], and *Listeria* adhesion protein, which interacts with *Hsp60* to promote bacterial adhesion to intestinal epithelial cells [60,61]. In addition, some capsule biosynthesis-related genes are present in DSM 2950Z, such as those for *Capsule I*, which play significant roles in immune evasion and contribute to disease pathogenesis [62].

GIs are classified as pathogenicity islands (PAIs), metabolic islands, resistance islands, or symbiotic islands [63]. PAIs contain genes involved in disease and can be a source of toxins that, under adverse conditions, contribute to pathogenicity [64]. Two GIs in DSM 2950 contain genes coding for toxins (RelE and antitoxin), and toxin-antitoxin genes that are present in *Bifidobacterium longum* subsp. *infantis* ATCC 15,697 allow these bifidobacteria to regulate growth, survival, and metabolism over a broad range of environmental stresses and can be used as functional metagenomic biomarkers [65]. Moreover, the toxin-antitoxin (TA) system acts as a regulator of many genes in pathogenic bacteria; for example, the expression of *Enterococcus* virulence genes is reduced by the mazET TA system [66].

Recently, increased antibiotic resistance has had a dramatic effect on global health. Thus, EFSA guidelines state that bacteria intended for human consumption must be subjected to antibiotic susceptibility assays to confirm their safety [67,68]. DSM 2950 is resistant to fluoroquinolone antibiotics such as ciprofloxacin, which is consistent with the fact that *L. fermentum* showed efflux pump-driven resistance to fluoroquinolones [67]. The resistance of lactic acid bacteria to aminoglycoside antibiotics such as neomycin and kanamycin is considered to be intrinsic, as they produce a variety of enzymes that degrade and thus deactivate antibiotics [69]. Consistent with this, DSM 2950 was highly tolerant of kanamycin and neomycin, due to its antibiotic efflux and inactivation mechanisms. Cauwerts et al. [70] found that chicken *Lactobacillus* strains carried the *ermC* gene but did not show resistance to macrolide or lincosamide, which is consistent with our results. Resistance in most probiotics can be acquired through HGT, and the gastrointestinal tract is a complex ecosystem that facilitates the exchange of resistance genes between microbiota (both commensal and pathogenic) [71,72]. Vancomycin-resistant enterococci (VRE) are recognized as an important cause of nosocomial infections due to their ability to acquire antibiotic resistance genes, and have gradually become a challenging threat in human medicine [73]. Genotypic studies identified som*e vancomycin-tolerant genes* (e.g., *vanHA*, *vanHB*, *vanRE*, *vanRF*, *vanRG*, *vanRM*, *vanRN*) in DSM 2950. The genes that encode d-Ala:d-Lac ligases, such as *vanA* and *vanB*, often result in high-level vancomycin resistance. Meanwhile, the genes that encode D-Ala:D-Ser ligases, including *vanE*, *vanG*, *vanM*, and *vanN*, generally result in low-level resistance [74]. Previous research has shown that the van genes for types A, B, D, and E are not endogenous and all appear to be acquired [75]; Leclercq et al. [76] found that vancomycin resistance in VRE was mediated by transposons on plasmids. In fact, the DSM2950 genome does not carry a plasmid and the results of phenotypic experiments show that DSM2950 is sensitive to vancomycin. *blaI*, *blaR1* and *blaR2* are responsible for regulating the expression of β-lactamase and contribute to the inactivation of β-lactam antibiotics [77]. However, DSM 2950 did not perform resistance to β-lactam antibiotics such as Ampicillin. Resistance genes are usually present on mobile genetic elements, such as plasmids, transposons, or integrins, and thus, we speculate that the inconsistency between phenotypic and genotypic results may be due to bacteria carrying resistance genes without expressing them.

The hemolytic characteristics of bacteria may cause cell lysis and contribute to the breakdown of hemoglobin to release bound iron [78]. According to the safety assessment guidelines of the Food and Agriculture Organization of the United Nations and the World Health Organization, hemolytic activity is a significant indicator of toxicity [79]. In our hemolysis testing, a hemolytic ring did not form around the microbial colonies of DSM 2950, which indicated that DSM 2950 was not capable of hemolysis.

Acute oral toxicity was proposed as a basic test for assessing the safety of new potential probiotics [80]. In this context, daily oral administration of 2.5 × 10^9^, 5 × 10^10^, or 2.5 × 10^12^ CFU/kg body weight/day lactic acid bacteria (*L. rhamnosus* HN001, *L. acidophilus* HN017 or *B. lactis* HN019) for 4 weeks was found to have no adverse effects on the health of BALB/c mice [34]. Consistent with this, our experimental results indicated that daily DSM 2950 doses of 1 × 10^9^ and 1 × 10^10^ CFU/kg body weight did not adversely affect the general health status of male or female mice, which suggests that approximately 3.5 × 10^13^ CFU doses of live DSM 2950 cells are safe for 70 kg healthy adult humans. This also confirmed that this strain possesses the potential to be safe for human consumption.

The hematopoietic system is one of the most sensitive parameters for assessing drug toxicity in humans and animals, and abnormalities in serum parameters such as ALT, AST, and ALP may indicate liver damage [81]. In our study, no significant difference was detected between the control and treatment groups, indicating that oral administration of DSM 2950 for a period of 14 days does not adversely affect the hematological and blood biochemical parameters of mice.

The liver is the main organ responsible for metabolism and thus reflects the health status of the body, the spleen is the human body’s largest immune organ and is involved in the immune response in body fluids and cells, and the intestinal mucosa is a barrier to the penetration of toxins and substances generated by potential pathogens [82,83]. We found that the morphology of liver, spleen, kidney, and colon tissues of mice treated with different doses of DSM 2950 were normal and free of lesions, suggesting that DSM 2950 was non-toxic and non-pathogenic.

## 5. Conclusions

Given the properties of *Blautia* and the potential utility of the *Blautia* strain DSM 2950 strain to relieve inflammation, we performed multifaceted safety assessments of DSM 2950 that involved genotypic assays (to detect virulence genes, GIs and antibiotic resistance genes), phenotypic assays (to detect antibiotic resistance and hemolytic activity), and acute toxicity tests to demonstrate it does not raise safety concerns and possesses the potential to be safe for human consumption. However, this represents only a preliminary safety assessment of DSM 2950; further genotoxicity, sub-chronic toxicity, and teratogenicity tests should be performed to confirm its safety and to provide theoretical support for its use in food and pharmaceuticals. Moreover, safety assessments of other species and strains of this genus should also be performed.

## Figures and Tables

**Table 1:** Virulence genes identified in *Blautia producta* DSM 2950.

Class of Virulence Genes	Virulence Gene Function	Virulence Gene Name
Regulation of virulence-associated genes	Regulation	*PhoP*, *RelA*
Nonspecific virulence gene	Iron uptake system	*FbpABC*, *FeoAB*, *HitABC*
Offensive virulence gene	Secretion system	*Dot*/*Icm*, *HSI-I*,
	Adherence	*PI-2*, *PI-2a*, *Lap*, *LPS*, *FbpA*, *Hyaluronic acid capsule*, *Hsp60*
Defensive virulence gene	Stress protein	*MsrAB*, *KatA*, *ClpC*, *ClpP*
	Antiphagocytosis	*Capsule*, *Capsule I*, *Alginate*
Unclassified virulence gene	_	*Beta-hemolysin*/*cytolysin*, *BSH*, *Cytolysin*, *Enterobactin*, *Hemolysin*, *LOS*, *MgtBC*, *mycobactin*, *peritrichous flagella*, *PgdA*, *Phenazines biosynthesis*, *type IV pili*, *Urease*

**Table 2:** Antibiotic susceptibility test of *Blautia producta* DSM 2950.

Antibiotics (μg/mL)	DSM 2950	ATCC 334	Cut-Off Values
Kanamycin	MIC < 256	MIC < 64	MIC ≤ 64
Streptomycin	MIC < 64	MIC < 32	MIC ≤ 64
Neomycin	MIC < 64	MIC < 8	MIC ≤ 32
Tetracycline	MIC < 0.125	MIC < 2	MIC ≤ 4
Erythromycin	MIC < 0.25	MIC < 0.5	MIC ≤ 1
Clindamycin	MIC < 8	MIC < 0.5	MIC ≤ 1
Ampicillin	MIC < 1	MIC < 1	MIC ≤ 4
Amoxicillin	MIC < 0.5	MIC < 1	MIC ≤ 2
Trimethoprim	MIC < 32	Resistance	MIC ≤ 32
Ciprofloxacin	MIC < 32	MIC < 4	MIC ≤ 4
Chloramphenicol	MIC < 4	MIC < 8	MIC ≤ 4
Rifampin	MIC < 0.125	MIC < 0.125	MIC ≤ 2
Vancomycin	MIC < 4	Resistance	MIC ≤ 4

**Table 3:** Antibiotic resistance genes of *Blautia producta* DSM 2950.

ARO Category	Antibiotic Resistance Gene	Resistance Mechanism
efflux pump	*bcrA*, *drrA*, *lmrD*, *novA*, *oleC*, *sav1866*	-
macrolide antibiotic	*mtrA*, *macB*	antibiotic efflux
fluoroquinolone antibiotic	*arlR*, *CdeA*, *efrA*, *patB*	antibiotic efflux
clindamycin antibiotic	*lsaB*	antibiotic target protection
aminoglycoside antibiotic	*baeR*, *kdpE*	antibiotic efflux
	*APH (3′)-IIIa*, *ANT (6)-Ia*	antibiotic inactivation
glycopeptide antibiotic	*vanHA*, *vanHB*, *vanRA*, *vanRG*, *vanRM*, *vanRN*, *vanSA*, *vanTG*,	antibiotic target alteration
	*vanRE*, *vanRF*, *vanRI*, *vanU*	-
streptogramin antibiotic	*vatF*	antibiotic inactivation
tetracycline antibiotic	*rpsJ*	antibiotic target protection
peptide antibiotic	*bacA*, *PmrA*, *PmrE*	antibiotic target alteration
	*blaI*, *blaR1*	-

**Table 4:** Effect of *Blautia producta* DSM 2950 on hematological parameters.

Parameter	Control-Female	10^9^-Female	10^10^-Female	Control-Male	10^9^-Male	10^10^-Male
WBC (10^9^/L)	2.40 ± 0.47	2.97 ± 0.25	2.79 ± 0.43	2.58 ± 0.46	3.66 ± 0.89	2.58 ± 0.62
Neu (10^9^/L)	0.24 ± 0.13	0.28 ± 0.06	0.41 ± 0.15	0.25 ± 0.06	0.39 ± 0.16	0.35 ± 0.12
Lym (10^9^/L)	2.26 ± 0.35	2.61 ± 0.28	2.47 ± 0.26	1.91 ± 0.23	2.49 ± 0.44	2.10 ± 0.50
Mon (10^9^/L)	0.05 ± 0.07	0.05 ± 0.04	0.05 ± 0.08	0.03 ± 0.01	0.03 ± 0.02	0.04 ± 0.04
Eos (10^9^/L)	0.03 ± 0.01	0.02 ± 0.01	0.03 ± 0.01	0.04 ± 0.03	0.03 ± 0.01	0.05 ± 0.01
Bas (10^9^/L)	0.00 ± 0.00	0.00 ± 0.00	0.01 ± 0.01	0.00 ± 0.00	0.02 ± 0.02	0.00 ± 0.00
Neu (%)	11.44 ± 3.03	9.14 ± 1.62	10.6 ± 1.70	11.82 ± 2.49	13.32 ± 1.93	13.48 ± 2.33
Lym (%)	83.82 ± 5.73	87.64 ± 3.77	86.28 ± 2.16	80.54 ± 10.09	69.54 ± 11.67	81.62 ± 2.92
Mon (%)	2.70 ± 3.19	2.78 ± 4.89	1.84 ± 1.55	1.70 ± 0.70	3.08 ± 3.11	2.78 ± 3.23
Eos (%)	0.20 ± 0.22	0.24 ± 0.11	0.28 ± 0.16	0.40 ± 0.14	0.40 ± 0.16	0.42 ± 0.22
Bas (%)	0.24 ± 0.09	0.20 ± 0.10	0.28 ± 0.18	0.14 ± 0.15	0.30 ± 0.12	0.20 ± 0.12
RBC (10^12^/L)	7.91 ± 0.59	7.72 ± 0.55	7.38 ± 0.42	7.84 ± 0.27	7.41 ± 0.23	7.40 ± 0.35
HGB (g/L)	104 ± 17.83	100 ± 4.80	109.20 ± 11.30	105.80 ± 8.38	110.40 ± 6.58	107.00 ± 6.89
HCT (%)	40.14 ± 4.44	35.76 ± 4.53	38.38 ± 2.65	39.19 ± 1.98	40.56 ± 1.22	41.62 ± 1.85
MCV (fL)	43.34 ± 0.77	43.9 ± 1.32	42.4 ± 1.44	41.90 ± 1.70	43.90 ± 1.59	42.94 ± 0.92
MCH (pg)	15.07 ± 0.29	15.10 ± 0.51	14.94 ± 0.69	15.30 ± 0.26	14.80 ± 1.09	15.02 ± 0.94
MCHC (g/L)	333.40 ± 9.29	328.60 ± 12.82	326.20 ± 6.46	330.72 ± 5.71	330.92 ± 13.06	337.14 ± 6.56
RDW-CV (%)	12.40 ± 0.22	12.60 ± 0.29	12.32 ± 0.36	12.50 ± 0.16	12.26 ± 0.24	12.50 ± 0.23
RDW-SD (fL)	27.52 ± 0.49	26.85 ± 1.78	27.46 ± 0.29	27.66 ± 0.49	27.83 ± 0.75	26.79 ± 0.39
PLT (10^9^/L)	972.22 ± 51.26	991.69 ± 90.82	1043.00 ± 117.1	819.51 ± 91.73	762.73 ± 62.48	839.79 ± 75.86
MPV (fL)	4.70 ± 0.14	4.96 ± 0.15	5.00 ± 0.62	5.04 ± 0.96	4.82 ± 1.11	4.56 ± 0.05
PDW (%)	12.66 ± 0.05	12.68 ± 0.08	12.84 ± 0.27	12.54 ± 0.17	12.78 ± 0.51	12.72 ± 0.11
PCT (%)	0.46 ± 0.05	0.43 ± 0.02	0.44 ± 0.03	0.37 ± 0.03	0.36 ± 0.04	0.34 ± 0.02

Data were presented as means ± SD of measurements per group (*n* = 5) for each parameter. *p* < 0.05 was considered statistically significant, while *p* < 0.01 was considered extremely statistically significant. Abbreviations: WBC, white blood cells; Neu, neutrophils; Lym, lymphocytes; Mon, monocytes; Eos, eosinophils; Bas, basophils; RBC, red blood cells; HGB, hemoglobin concentration; HCT, hematocrit value; MCV, mean corpuscular volume; MCH, mean corpuscular hemoglobin; MCHC, mean corpuscular hemoglobin concentration; RDW, red cell distribution width; CV, coefficient of variation; SD, standard deviation; PLT, platelet; MPV, mean platelet volume; PDW, platelet distribution width; PCT, thrombocytocrit.

**Table 5:** Effect of *Blautia producta* DSM 2950 on serum biochemical parameters.

Parameter	Control-Male	10^9^-Male	10^10^-Male	Control-Female	10^9^-Female	10^10^-Female
Glu (mmol/L)	3.70 ± 0.87	3.07 ± 0.39	3.09 ± 0.55	3.04 ± 0.34	3.03 ± 0.33	2.99 ± 0.51
TC (mmol/L)	2.74 ± 0.23	3.16 ± 0.46	2.86 ± 0.09	1.86 ± 0.23	1.74 ± 0.26	1.92 ± 0.20
TG (mmol/L)	1.33 ± 0.35	1.29 ± 0.23	1.36 ± 0.12	0.92 ± 0.12	0.86 ± 0.11	0.89 ± 0.14
ALT(U/L)	26.62 ± 4.17	29.50 ± 3.49	27.85 ± 2.47	31.50 ± 3.15	28.56 ± 1.78	28.53 ± 3.87
AST(U/L)	135.60 ± 19.29	126.96 ± 13.00	138.72 ± 23.85	151.68 ± 68.55	143.46 ± 36.60	153.66 ± 41.73
ALP(U/L)	174.00 ± 18.85	163.20 ± 29.29	154.20 ± 9.86	178.80 ± 27.13	202.80 ± 22.21	186.60 ± 21.15
γ-GT (U/L)	3.06 ± 0.68	2.88 ± 0.40	3.06 ± 0.83	2.70 ± 0.56	3.30 ± 0.60	2.88 ± 1.11
CHE(U/L)	4714.20 ± 469.40	4749.00 ± 347.61	4956.00 ± 390.65	6661.80 ± 1085.12	6528.00 ± 393.56	6400.20 ± 802.77
TP(g/L)	57.42 ± 3.68	52.98 ± 2.44	56.82 ± 2.57	53.40 ± 2.96	55.74 ± 2.89	54.48 ± 5.53
ALB(g/L)	35.64 ± 2.04	32.58 ± 2.44	34.92 ± 2.15	34.50 ± 2.34	36.18 ± 1.96	34.98 ± 4.13
CK(U/L)	1012.27 ± 229.25	785.61 ± 236.92	990.99 ± 233.37	716.28 ± 479.39	814.98 ± 308.76	943.80 ± 306.41
LDH(U/L)	370.20 ± 61.02	337.32 ± 18.91	324.18 ± 43.42	390.9 ± 158.67	358.74 ± 98.81	377.88 ± 141.29

Data were presented as means ± SD of measurements per group (*n* = 5) for each parameter. *p* < 0.05 was considered statistically significant (*), while *p* < 0.01 was considered extremely statistically significant (**). Abbreviations: Glu, glucose; TC, total cholesterol; TG, triglyceride; ALT, alanine aminotransferase; AST, aspartate aminotransferase; ALP, alkaline phosphatase; γ-GT, γ-glutamyl transpeptidase; CHE, cholinesterase; TP, total protein; ALB, albumin; CK, creatine kinase; LDH, lactic dehydrogenase.

## Data Availability

The draft genome sequence of *Blautia producta* DSM 2950 is available at the GenBank of the NCBI database (accession number SRR14193994).

## Supplementary Materials

The following are available online at https://www.mdpi.com/article/10.3390/microorganisms9050908/s1, Table S1. Animal experimental design. Table S2. Genomic islands identified in *Blautia producta* DSM 2950. More representative pictures of the organs after hematoxylin-and-eosin-staining are provided as the supplementary figures.
