# Supplementary material for: A Comprehensive Assessment of the Safety of Blautia producta DSM 2950
Source: Microorganisms. 2021 Apr 23;9(5):908. doi: 10.3390/microorganisms9050908 (PMC8146736; doi:10.3390/microorganisms9050908)
Supplement: Supplementary file 1 [file microorganisms-09-00908-s001.zip › microorganisms-1168692-supplementary.pdf]

Table S1. Animal experimental design.

Group	Sex	Mice Number	Gavage substance	Volume
Control-male	male	n=5	10% wt/v skimmed milk	200 µl
10^9-male	male	n=5	DSM 2950 5×10^9 CFU/ml	200 µl
10^{10}-male	male	n=5	DSM 2950 5×10^{10} CFU/ml	200 µl
Control-female	female	n=5	10% wt/v skimmed milk	200 µl
10^9-female	female	n=5	DSM 2950 5×10^9 CFU/ml	200 µl
10^{10}-female	female	n=5	DSM 2950 5×10^{10} CFU/ml	200 µl

Table S2. Genomic islands identified in Blautia producta DSM 2950

Island ID	Genes in Island	Size（Kb）	Main functional genes within island
GI01	gene0285-gene0302	14.6	Recombinase(g0285), transposase(g0293), ATPase AAA(g0295), integrase(g0296), glutamine amidotransferase(g0300)
GI02	gene0442-gene0468	20.2	glycosyl transferase family 1(g0442), radical SAM protein(g0445), Glycosyltransferases(g0447), phage tail tape measure protein(g0459)
GI03	gene0782-gene0842	50.7	mobilization protein(g0784), DNA topoisomerase III(g0790), bacteriocin(g0809), conjugal transfer protein TraE(g0814), TraG(g0824), stage V sporulation protein G(g0817), membrane protein(g0818), Maff2 family(g0821), toxin RelE(g0835)
GI04	gene2085-gene2121	37.8	plasmid segregation centromere-binding protein ParR(g2102), integrase(g2110), DNA processing protein DprA(g2113), type II secretion protein F(g2120)
GI05	gene2966-gene2978	15.4	SIR2 family protein(g2967), serine recombinase(g2969), N-acetylmuramoyl-L-alanine amidase(g2971), holing(g2972),
GI06	gene2991-gene3019	13.3	Phage Terminase(g2994), gamma glutamylcyclotransferase(g3002), virulence associated protein(g3003), HrgA protein(g3013)
GI07	gene3704-gene3722	15.4	sugar ABC transporter permease(g3707), 50S ribosomal protein L33(g3710), GHKL domain-containing protein(g3715)
GI08	gene4522-gene4545	22.1	membrane protein(g4530), conjugal transfer protein TraE(4532), TraG(g4527),bacterial mobilization protein MobC(4545), antitoxin(g4528)
GI09	gene4681-gene4691	11.9	two-component sensor histidine kinase(4683), transposase(g4685), MmcQ protein(g4686), TraE family protein(g4689)
GI10	gene4863-gene4876	13.1	two-component sensor histidine kinase(g4869), peptide ABC transporter ATP-binding protein(g4870), DNA-directed RNA polymerase sigma-70 factor(g4873)

Colon

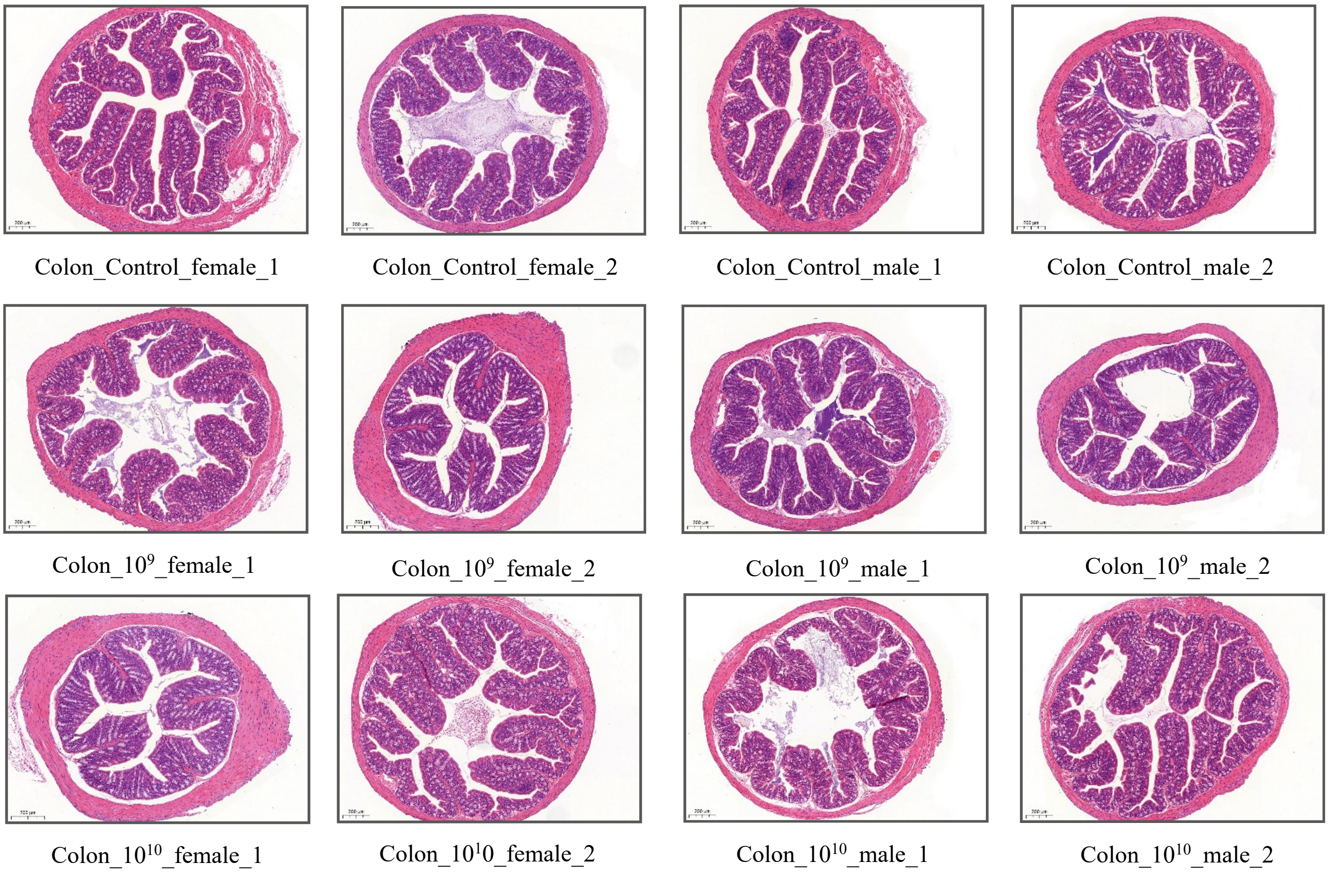
Colon_Control_female_1
Colon_Control_female_2
Colon_Control_male_1
Colon_Control_male_2
Colon_10^{9}_female_1
Colon_10^{9}_female_2
Colon_10^{9}_male_1
Colon_10^{9}_male_2
Colon_10^{10}_female_1
Colon_$10^{1}0$_female_2
Colon_10^{10}_male_1
Colon_10^{10}_male_2

Kidney

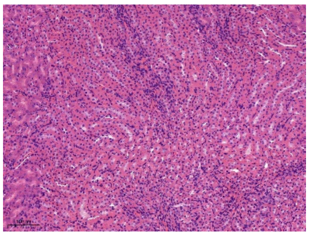

Kidney_Control_female_1

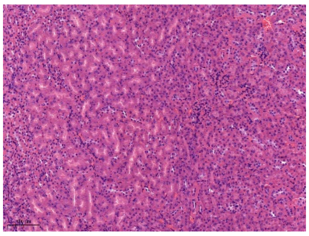

Kidney_Control_female_2

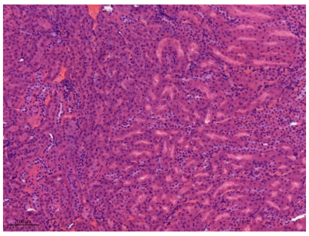

Kidney_Control_male_1

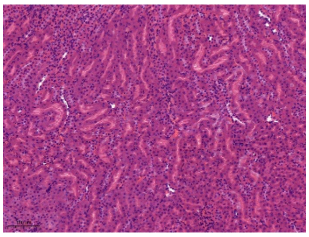

Kidney_Control_male_2

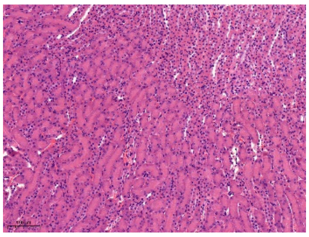

Kidney_10^{9}_female_1

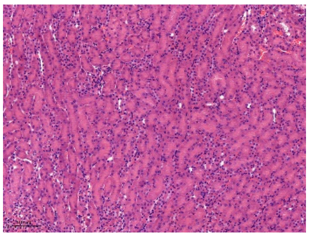

Kidney_10^{9}_female_2

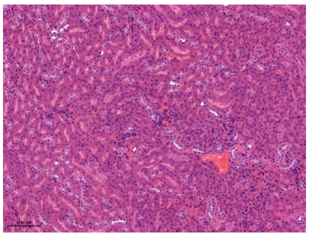

Kidney_10^{9}_male_1

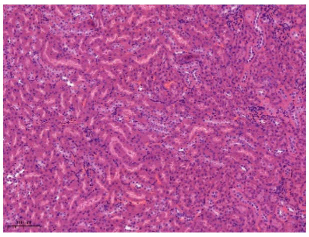

Kidney_10^{9}_male_2

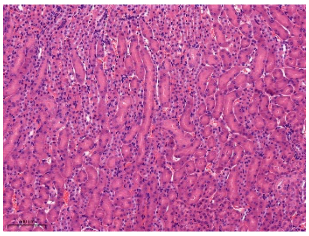

Kidney_10^{10}_female_1

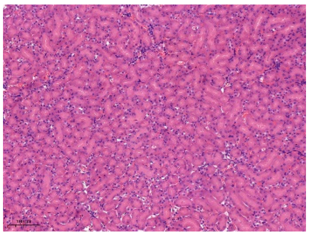

Kidney_10^{10}_female_2

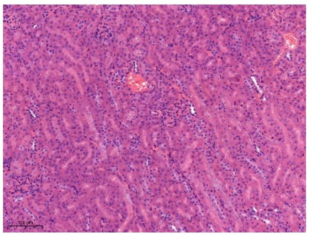

Kidney_10^{10}_male_1

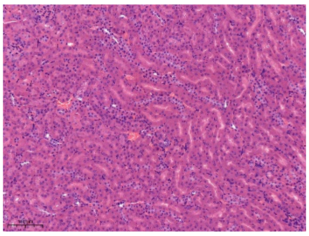

Kidney_10^{10}_male_2

Liver

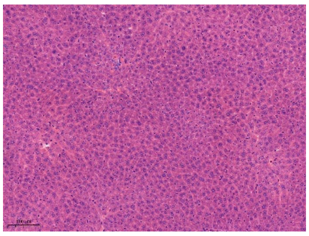

Liver_Control_female_1

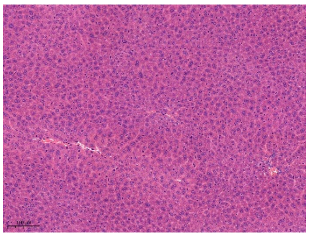

Liver_Control_female_2

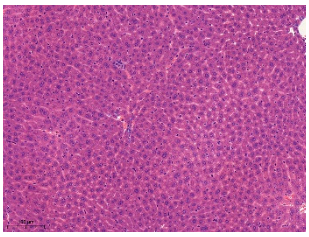

Liver_Control_male_1

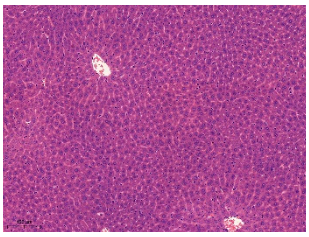

Liver_Control_male_2

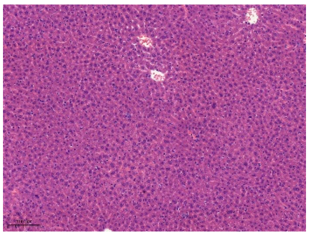

Liver_10^9_female_1

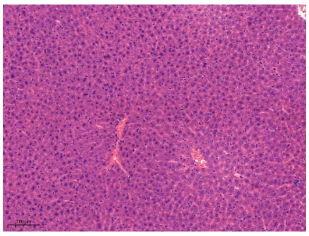

Liver_10^9_female_2

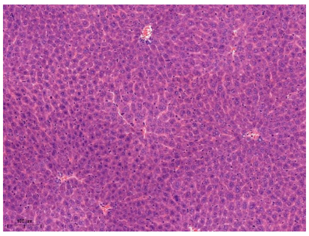

Liver_10^9_male_1

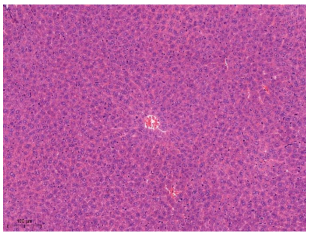

Liver_10^9_male_2

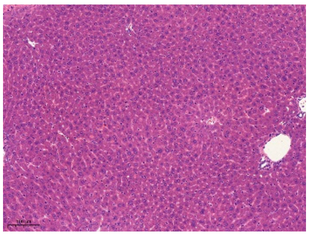

Liver_10^{10}_female_1

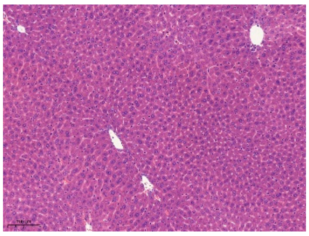

Liver_10^{10}_female_2

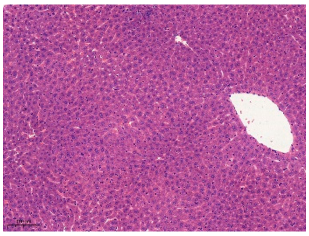

Liver_10^{10}_male_1

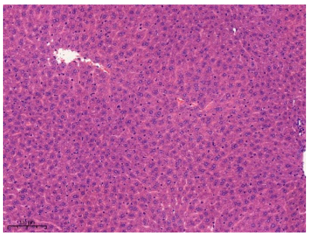

Liver_10^{10}_male_2

Spleen

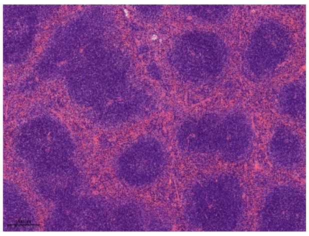

Spleen_Control_female_1

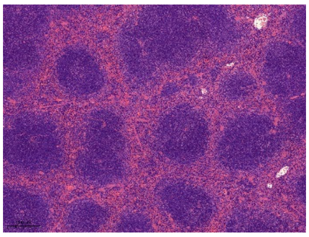

Spleen_Control_female_2

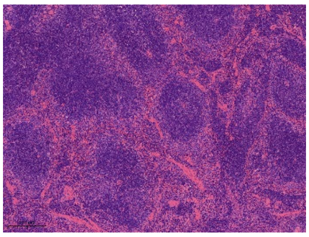

Spleen_Control_male_1

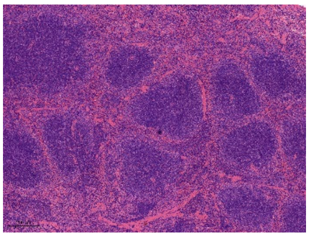

Spleen_Control_male_2

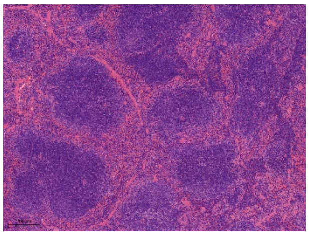

Spleen_10^9_female_1

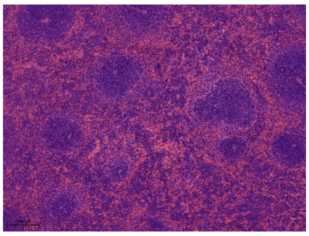

Spleen_10^9_female_2

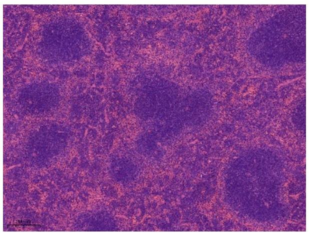

Spleen_10^9_male_1

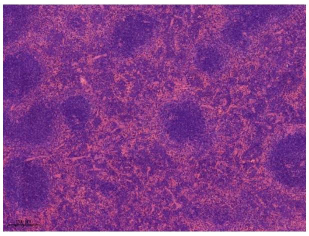

Spleen_10^9_male_2

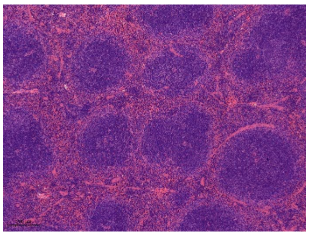

Spleen_10^{10}_female_1

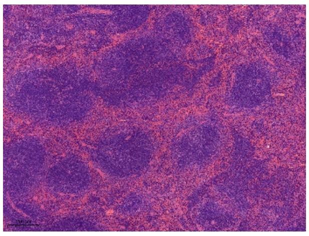

Spleen_10^{10}_female_2

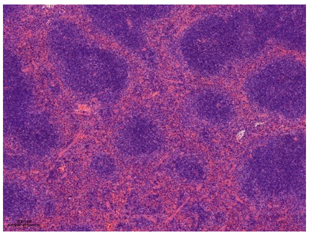

Spleen_10^{10}_male_1

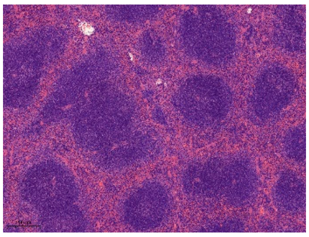

Spleen_10^{10}_male_2
